# Transforming geographic scale: a comparison of combined population and areal weighting to other interpolation methods

**DOI:** 10.1186/s12942-017-0102-z

**Published:** 2017-08-07

**Authors:** Elaine Hallisey, Eric Tai, Andrew Berens, Grete Wilt, Lucy Peipins, Brian Lewis, Shannon Graham, Barry Flanagan, Natasha Buchanan Lunsford

**Affiliations:** 1 Agency for Toxic Substances and Disease Registry, Geospatial Research, Analysis, and Services Program, Centers for Disease Control and Prevention, 4770 Buford Highway, MS F09, Atlanta, GA 30341-3717 USA; 2 Division of Cancer Prevention and Control, Centers for Disease Control and Prevention, Atlanta, GA USA

**Keywords:** Areal interpolation, Areal weighting, Population weighting, Disaggregation, Geographic scale, Adolescent cancer, Compressed Mortality File

## Abstract

**Background:**

Transforming spatial data from one scale to another is a challenge in geographic analysis. As part of a larger, primary study to determine a possible association between travel barriers to pediatric cancer facilities and adolescent cancer mortality across the United States, we examined methods to estimate mortality within zones at varying distances from these facilities: (1) geographic centroid assignment, (2) population-weighted centroid assignment, (3) simple areal weighting, (4) combined population and areal weighting, and (5) geostatistical areal interpolation. For the primary study, we used county mortality counts from the National Center for Health Statistics (NCHS) and population data by census tract for the United States to estimate zone mortality. In this paper, to evaluate the five mortality estimation methods, we employed address-level mortality data from the state of Georgia in conjunction with census data. Our objective here is to identify the simplest method that returns accurate mortality estimates.

**Results:**

The distribution of Georgia county adolescent cancer mortality counts mirrors the Poisson distribution of the NCHS counts for the U.S. Likewise, zone value patterns, along with the error measures of hierarchy and fit, are similar for the state and the nation. Therefore, Georgia data are suitable for methods testing. The mean absolute value arithmetic differences between the observed counts for Georgia and the five methods were 5.50, 5.00, 4.17, 2.74, and 3.43, respectively. Comparing the methods through paired t-tests of absolute value arithmetic differences showed no statistical difference among the methods. However, we found a strong positive correlation (r = 0.63) between estimated Georgia mortality rates and combined weighting rates at zone level. Most importantly, Bland–Altman plots indicated acceptable agreement between paired arithmetic differences of Georgia rates and combined population and areal weighting rates.

**Conclusions:**

This research contributes to the literature on areal interpolation, demonstrating that combined population and areal weighting, compared to other tested methods, returns the most accurate estimates of mortality in transforming small counts by county to aggregated counts for large, non-standard study zones. This conceptually simple cartographic method should be of interest to public health practitioners and researchers limited to analysis of data for relatively large enumeration units.

## Background

The challenge of transforming spatial data collected at one scale to another scale, often referred to as areal interpolation or cross-area estimation, has long been recognized in spatial analysis [1]. In many cases, geographic boundaries, such as counties, are unsuitable in terms of the units needed for meaningful data analysis. This spatial misalignment of data is referred to as the change-of-support problem, which is concerned with inferences about the value of any particular variable at an enumeration unit different from that at which data were collected [2, 3]. Researchers and practitioners sometimes require estimates for non-standard geographic areas, i.e. target zones, to be derived from existing source zones, i.e. the zones from which the data are obtained. For example, an analyst who requires data for a non-standard enumeration unit, say a zone surrounding a U.S. hospital (target zone), must transform data collected at another zone level, such as a group of U.S. census tracts (source zones), to match the boundaries of the zone surrounding the hospital. With the growth of available data and geographic information systems that can integrate these data, there has been a parallel increase in the development of methods to address this problem.

Geospatial techniques, well documented in texts and the literature, are widely used to deal with transformation between scales [1–8]. Examples of methods include centroid assignment, areal weighting, dasymetric, regression, and geostatistical (or surface-generating).

For simple geographic centroid assignment, counts of some phenomenon are summed for a source zone, and allocated to the geographic centroid, that is, the areal center of gravity of the zone [9, 10]. Values assigned to zone centroids that fall within a target zone are then summed to estimate a count for the target zone. The binary nature of this technique means centroid assignment is either completely in or out of the zone, in other words, an all-or-nothing operation. Additionally, the geometry of the zone’s polygon affects the positioning of the geographic centroid. Automated centroid placement is likely to be different depending upon the selection of input zone polygons.

Areal weighting, often used to disaggregate populations, is a cartographic overlay method that preserves volume, meaning subdivided populations sum to the original population. Weights are determined from the size of the overlapping source and target zone areas. For example, if a source zone (e.g., a census tract) with a population of 4000 is split so that 25% of the area falls in target zone A, and 75% falls in target zone B, 1000 individuals are allocated to target zone A and 3000 individuals to target zone B. A limitation is that areal weighting assumes an even distribution of population within each source zone [6, 8].

Methods exist to estimate prospective error in areal weighting and, as they are relevant to this paper, are discussed here. Simpson describes two measures to express the amount of estimation involved in the transformation from source to target zones: the degree of hierarchy, and the degree of fit [11]. The degree of hierarchy, or nesting, for an entire study area is the proportion of all source zones that fall completely within any of the target zones. The degree of hierarchy for an individual target zone is the proportion of source zones that fall completely within that target zone. Degree of hierarchy is calculated as:

1 $$H = \left( {\frac{{\mathop \sum \nolimits_{s,t} \left( {w_{st} = 1} \right)}}{{\mathop \sum \nolimits_{s} \left( 1 \right)}}} \right)$$

where: *H* is the degree of hierarchy; *s* is a source zone; *t* is a target zone; and *w*_*st*_ is the areal overlap of the source zone with the target zone.

The degree of fit, or overlap, for the entire study area sums the maximum proportion, or weight, of each source zone as a proportion of all source zones. The degree of fit for a single target zone sums the weights of each source zone as a proportion of all source zones within the target zone. Degree of fit is calculated as:

2 $$F = \left( {\frac{{\mathop \sum \nolimits_{s} \left( {\hbox{max} \, w_{st} } \right)}}{{\mathop \sum \nolimits_{s} \left( 1 \right)}}} \right)$$

where: *F* is the degree of fit; *s* is a source zone; *t* is a target zone; and *w*_*st*_ is the areal overlap of the source zone with the target zone.

Degree of hierarchy and degree of fit are usually multiplied by 100 to be expressed as percentages. The closer the output of these measures to 100%, the better the transformation estimate; accuracy increases as nesting increases and as the number of target zones decreases [12]. Researchers and practitioners, particularly in population geography, have used Simpson’s measures to estimate potential error in cartographic areal interpolation [13, 14].

Dasymetric techniques use various ancillary data, such as cadastral, land cover, remotely-sensed, or fine resolution population data, to inform data disaggregation [15–22]. Applying a process conceptually similar to a dasymetric approach in the first step of their population-weighted interpolation, Wilson and Mansfield transformed county-level mortality rates to congressional districts (CDs) [18]. They used ancillary population data at census block level, census blocks nesting completely within both counties and CDs. For each county, the researchers first assigned the same mortality rate to each of the census blocks within the county. They then multiplied each block rate by block population count as a proportion of the total CD population and finally summed all the population-weighted block rates to estimate a CD mortality rate. As well as improving area-to-area transformation, ancillary data can, for instance, also be applied to point-level data to generate population-weighted centroids.

The cartographic methods described above have generally been used to transform *large* populations and rates. However, regression and geostatistical methods can accommodate small counts as well. Global or regional regression approaches use ancillary data as explanatory variables to develop models that predict population distribution in the source zones to better estimate populations in the target zones. These models assume a relationship exists among the population and other variables, such as land cover or parcel data [6, 8, 23]. Regression models offer the ability to refine estimates with the incorporation of covariates and to measure uncertainty. However, they also introduce complexity [22], require transformation of covariate geography, and generally do not handle changing relationships across space, i.e., non-stationarity, as well as do dasymetric methods, for which estimates are locally fitted to each source zone [6].

Geostatistical methods are used to model spatial data to produce estimates where data are unavailable [2, 24–26]. Either a smooth prediction surface or a probability surface, created from points derived from source polygons, is aggregated back to target polygons. As with simple areal weighting, geostatistical analysis assumes smooth distribution changes across the landscape, which is not usually the case. In addition, building a valid model can be difficult, as complex geostatistical techniques are often applied inappropriately [27].

**Fig. 1:**
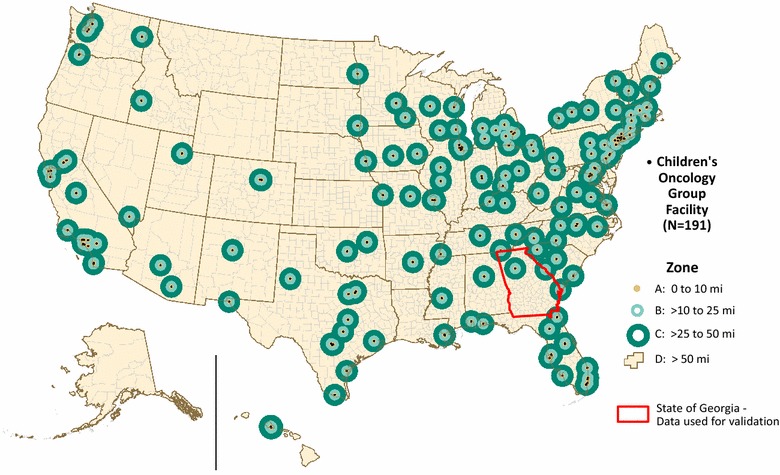
Children’s Oncology Group Institutions and Zones. The primary study encompasses the entire United States. This paper focuses on the validation of methods using Georgia adolescent cancer mortality data

The analysis discussed in this paper is part of a larger ecologic research project to determine a possible association between distance to pediatric cancer facilities and cancer mortality among adolescents, ages 15 through 19. Children’s Oncology Group (COG) institutions provide specialized cancer care for children through clinical trials and research. Whereas most children 14 years of age and younger are treated in a COG, the majority of adolescents are referred to adult oncology centers that have less access to clinical trials and thus less improvement in survival [28–30]. To examine mortality rates by sex, race, and ethnicity within zones at varying distances from these facilities we needed to estimate adolescent cancer mortality rates for four, multipart zones surrounding 191 COG facilities across the United States (Fig. 1). In this paper, we used Georgia adolescent cancer mortality data, examining mortality rates by sex by zone, to test the methods.

The four zones represent an effort to define each COG institution’s city core, an inner suburban ring, an outer suburban/exurban ring, and the balance of land beyond. Zone A encircles an area within 10 miles of any COG. Zones B and C are concentric rings with distances from a COG of >10 to 25 miles and >25 to 50 miles, respectively. Zone D comprises the remaining United States. Data available for the primary study included census tract level demographic data for rate denominators and U.S.-wide, county-level National Center for Health Statistics (NCHS) Compressed Mortality File (CMF) data for rate numerators. Although the tracts aggregate to counties, the four zones coincide with neither tracts nor counties. For this methods paper, we used residential address-level mortality data from the state of Georgia along with tract population data to evaluate methods to transform county mortalities (source zones), to the four study zones (target zones).

We sought to identify the simplest interpolation method that returned satisfactory mortality estimates. Given the large geographic scope of the primary research, i.e., zones encompassing the entire U.S., we aimed for straightforward methods with workable data requirements. In other words, we required a conceptually simple technique with readily available, statistically robust, nationwide data.

In this paper, we examine and discuss the results of five interpolation methods. Commonly used in research and practice, we explored geographic and population-weighted centroid assignment, simple areal weighting, and geostatistical areal interpolation. We also developed and tested a conceptually simple technique, combined population and areal weighting, which merges a dasymetric population weighting with areal weighting. We chose not to examine regression to estimate mortality because the sole intent of the primary study was to examine the association between adolescent cancer mortality and distance to a COG and we wanted to avoid the complexities of U.S.-wide regression models using multiple covariates. We believe cartographically-focused estimation techniques are more appropriate for this methods paper.

## Methods

Data sources for the primary study included U.S. Census 2000 and 2010 100% population counts at the tract level as well as 1999–2011 county-level cancer mortality data for those aged 15 through 19 from the NCHS CMF, which are compiled from individual state death certificates [31–33]. To preserve confidentiality, NCHS provides mortality data at the county level only, upon a substantiated request and signed data use agreement.^1^ However, some states consider death certificates public record and share residence-level point data. We therefore obtained point-level, adolescent cancer mortality data from Georgia, a state that releases mortality data for research, also upon a substantiated request and signed data use agreement, to assess the accuracy of our methods in this paper [34].

Inasmuch as the four COG study zones, A, B, C, and D, are independent of any standard enumeration unit, we estimated numerators and denominators for each zone. Numerator and denominator estimation were tied to census year because of the differing 2000 and 2010 geographies, particularly at the tract level. Though the census years fell at equal positions along the study’s time span of years 1999 through 2011, we could not “split” mortality data for the study’s mid-point year, 2005, because we did not have month of death. For that reason, we chose to use 7 years (1999 through 2005) of mortality data with Census 2000 geographies and populations and 6 years (2006 through 2011) of mortality data with Census 2010 geographies and populations. The mortality rate was calculated as the number of deaths over the 13-year study period for a specified population subgroup, such as males (numerator), divided by the total population, or person-years at risk, of that specific subgroup (denominator). We weighted the denominator population by census year:

3 $$13{ - }year\,death\;total/\left( {\left( {2000\,population*7} \right) + \left( {2010\;population*6} \right)} \right)$$

### Denominator (population count) estimation

For our testing, we estimated Georgia mortality for males, females, and the total population, aged 15 through 19. To approximate population for study zones surrounding a COG (i.e. zone A, B, C, and D) for the denominator, we used the Population Estimator tool, developed by CDC’s Geospatial Research, Analysis, and Services Program (GRASP), which performs simple areal weighting [35]. The area of overlap of the census tract (source zone) with the study zone was divided by the area of the entire census tract to obtain the proportion, or areal weight, of the tract area within the study zone. The population of interest for each tract (male, female, or overall) was then multiplied by the areal weight for that study zone as follows:

4 $$E_{pt} = \left( {\frac{{A_{zt} }}{{A_{t} }}} \right)*P_{t}$$

where: *E*_*pt*_ is the areal-weighted population estimate for the tract, or tract portion, within the study zone; *A*_*zt*_ is the geographic overlap area of the tract and study zone; *A*_*t*_ is the geographic area of the entire tract; and *P*_*t*_ is the tract population.

**Fig. 2:**
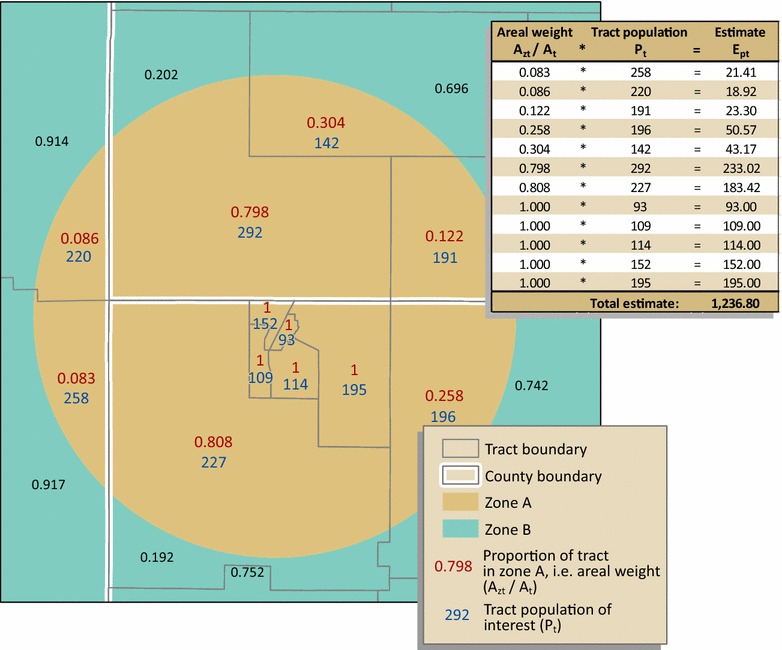
Denominator estimation for a hypothetical part of study zone A. The population for those aged 15 through 19 for each tract (*P*_*t*_) is multiplied by the proportion of the tract, or areal weight (*A*_*zt*_/*A*_*t*_), in the study zone. The output for each tract (*E*_*pt*_) in the entire zone is summed to obtain a population estimate for the study zone. *Note*: For graphic simplicity, only a subset of zones are shown in the figures. Methods are the same for each of the four study zones, A, B, C, and D

The resulting areal-weighted populations were summed to estimate a population total for the study zone for census years 2000 and 2010 (Fig. 2). We then calculated a weighted sum, as expressed in (3) above, to estimate a total 13-year population for the denominator. This process was repeated for each study zone, A, B, C, and D.

### Numerator (death count) estimation

Source zones for the numerator were counties with small numbers of deaths relative to the denominator populations. We tested five numerator estimation methods: (1) geographic centroid assignment, (2) population-weighted centroid assignment, (3) simple areal weighting, (4) combined population and areal weighting, and (5) geostatistical areal interpolation. For all five methods, we used Esri’s ArcGIS 10.3.1™ software. For the geostatistical method, we also used Esri’s Geostatistical Analyst extension in ArcMap.

#### Method 1: Geographic centroid assignment

**Fig. 3:**
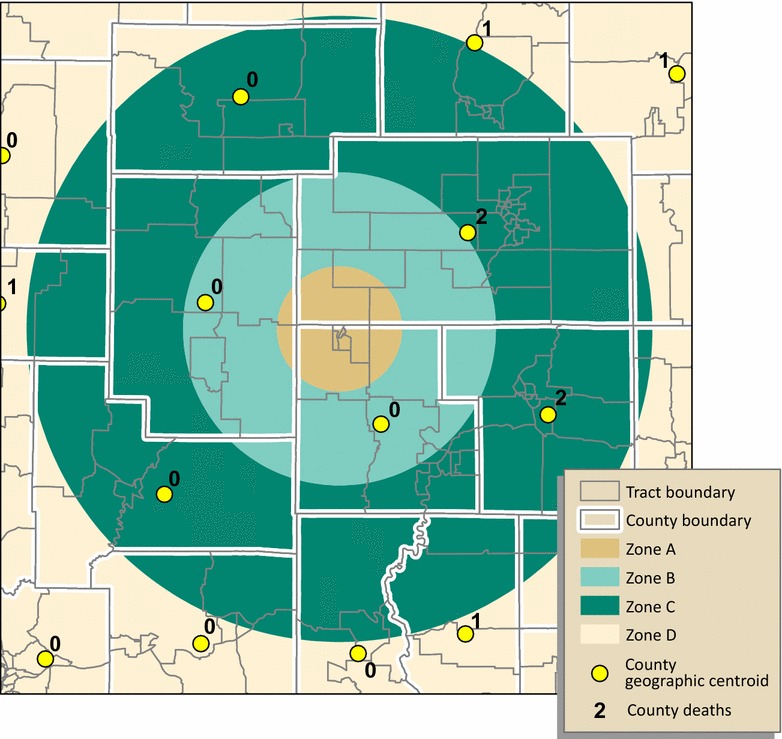
Geographic centroid assignment. Each county centroid is attributed a county mortality count for the population of interest. Mortality counts for centroids falling within each study zone are summed to estimate mortality, as a whole number, by zone. In this hypothetical example, zones A and B are assigned zero deaths, despite the overlap of three counties on zone A (two potential deaths) and five on zone B (four potential deaths). Zone C is assigned four deaths, but has the possibility of more

For geographic centroid assignment, we attributed Georgia Department of Public Health (GADPH) mortality counts to each county’s geographic centroid. County deaths assigned to centroids that fall within a study zone were summed, by sex and year, to estimate the number of deaths for that zone (Fig. 3).

#### Method 2: Population-weighted centroid assignment

**Fig. 4:**
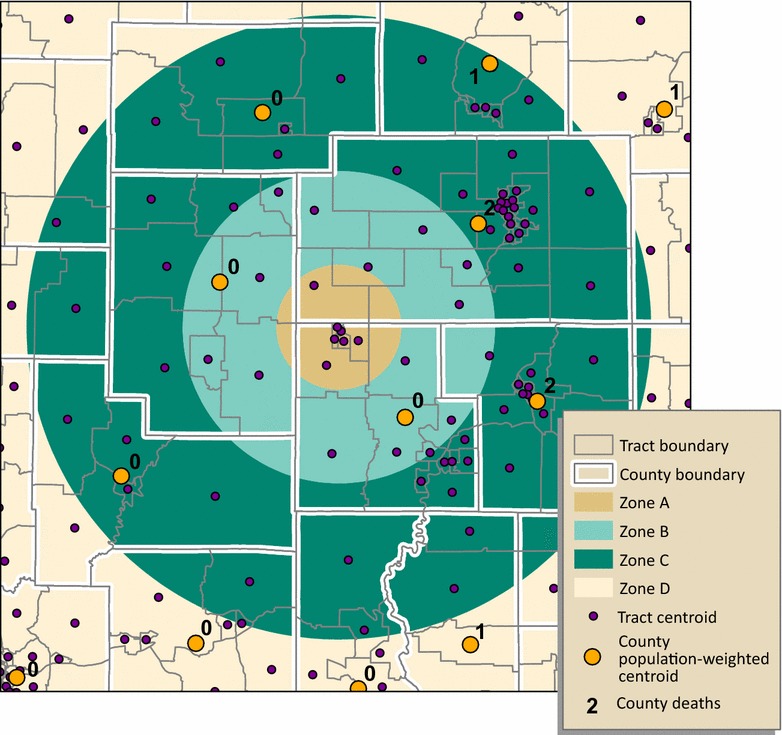
Population-weighted centroid assignment. Each tract centroid is attributed the population of interest. County centroids are placed using the mean center of tract centroids weighted by the tract population. Mortality counts for centroids falling within each study zone are summed to estimate mortality by zone. Results for zones A and B in this example, zero deaths for both, are the same as those for geographic centroid assignment. Zone C is assigned five deaths because the centroid in the northeast, with a value of “1,” is now positioned within zone C

For population-weighted centroid assignment, we attributed census tract populations of males and females aged 15 through 19, for years 2000 and 2010, to tract centroids. For each of Georgia’s 159 counties, we used the tract centroids to calculate mean centers, weighted by the tract-level population of interest, for each year. County deaths assigned to population-weighted centroids that fall within a study zone were summed, by sex and year, to estimate the number of deaths for that zone (Fig. 4).

#### Method 3: Simple areal weighting

Simple areal weighting, is the same technique used for the denominator estimates, as described above. In this case, the area of overlap of the *county* source zone with the COG target zone was divided by the area of the entire county to obtain the proportion, or areal weight, of the county area within the study zone. The number of deaths for each county was then multiplied by the corresponding areal weight for that county. The resulting areal-weighted mortalities were summed to estimate the number of deaths for the study zone. Figure 2 illustrates the method, with deaths by county source zones instead of population by tract source zones as shown.

#### Method 4: Combined population and areal weighting

To estimate numerators for each COG study zone we (1) used population weighting, a conceptually dasymetric approach similar to that of Wilson and Mansfield, to disaggregate mortality from county level counts to tract level estimates; then (2) weighted each tract mortality estimate by its geographic area within the study zone; and finally (3) aggregated the combined population- and areal-weighted tract mortality estimates for each zone. In contrast to Wilson and Mansfield, who used population-weighted interpolation to estimate *rates* for standard enumeration units (CDs), we estimated mortality *counts* using population subgroup proportions for non-standard study zones. In addition, unlike Wilson and Mansfield who transformed census blocks with 100% hierarchy and fit both from county and to CD, we performed the second step, areal weighting, because our non-standard COG target study zones split the source census tracts.

We detail the combined population and areal weighting process here. Because we had numbers of deaths by county-level only, we took advantage of the county/tract hierarchy and assigned each tract a population-weighted mortality estimate as follows:

5 $$E_{mt} = \left( {\frac{{P_{t} }}{{P_{c} }}} \right)*M_{c}$$

where: *E*_*mt*_ is the population-weighted mortality estimate for the tract; *P*_*t*_ is the tract population; *P*_*c*_ is the county population; and *M*_*c*_ is the number of deaths in the county.

**Fig. 5:**
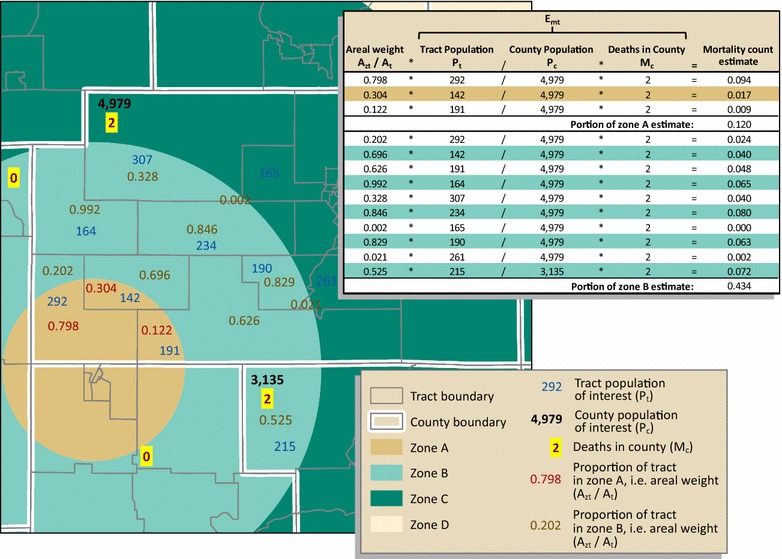
Combined population and areal weighting. The geographic area of the tract within the zone, the areal weight (*A*_*zt*_/*A*_*t*_), is multiplied by population-weighted mortality estimate for the tract (*E*_*mt*_). The output for each tract is then summed to estimate the number of deaths for the zone. We demonstrate, in this example, how estimates for portions of zones A and B are calculated. *Note*: As illustrated in Figs. 3 and 4, except for two counties, with two deaths each, the remaining counties within zones A and B recorded zero deaths for the population of interest; to simplify the illustration, we omitted counties with zero deaths. Also, because we show only portions of zones A and B, the estimates are technically only a portion of *M*_*z*_ for zones A and B

The output of Eq. (5) was multiplied by the geographic proportion of the tract that falls within the study zone, in other words, the areal weight (Fig. 5). This processing assumes an even distribution of tract population. We summed the resulting population and areal-weighted mortalities, by sex and year, to estimate the number of deaths for the zone. Expressed in its entirety, the study zone death count is estimated as:

6 $$M_{z} = \mathop \sum\nolimits_{{{\text{t}} = 1}}^{\text{n}} \left( {\frac{{ A_{zt} }}{{ A_{t} }}*E_{mt} } \right)$$

where: *M*_*z*_ is the study zone mortality count estimate; $\sum\nolimits_{{{\text{t}} = 1}}^{\text{n}} {}$ sums results for all tracts, or tract portions; *A*_*zt*_ is the geographic overlap area of the tract and study zone; *A*_*t*_ is the geographic area of the entire tract; and *E*_*mt*_ is the population-weighted mortality estimate for the tract.

#### Method 5: Geostatistical areal interpolation

To determine how geostatistical methods of interpolation compared to the cartographic methods described above, Georgia mortality counts were interpolated from county level data using one geostatistical interpolation model from among multiple explored, over-dispersed Poisson areal kriging, as described by Krivoruchko et al. [26], and implemented in ArcMap 10.3.1’s Geostatistical Wizard. We interpolated mortality count data for adolescent males and females separately. Using visual variography, we fitted a stable kriging interpolation model to a plot of empirical covariance versus distance, creating a continuous surface depicting the probability of event occurrence in the study area. The geostatistical method we used produced standardized root mean square error values of 1.02 for females and 1.12 for males, for which an ideal value would be 1.0. During variography we used a lattice spacing of 1000 m, a lag size of 5000 m, and 18 lags. The continuous probability surface was then used to estimate the mortality event counts for the COG zones, providing a numerator to determine a mortality rate for each zone based on the previously calculated population.

Statistical analyses to assess the methods included: (1) the distribution of county mortality counts, (2) measures of potential transformation error among numerator, denominator, and zones in terms of degrees of hierarchy and fit, and (3) absolute value arithmetic differences from observed Georgia mortality counts, t-tests on absolute value arithmetic differences among the five methods to check for statistical difference, Pearson’s r correlations between Georgia rates and estimated rates, and Bland–Altman plots depicting 95% level of agreement between Georgia mortality rates and those of the five methods [36–39].

## Results

### Distribution of adolescent cancer county mortality counts: Georgia versus the U.S

**Fig. 6:**
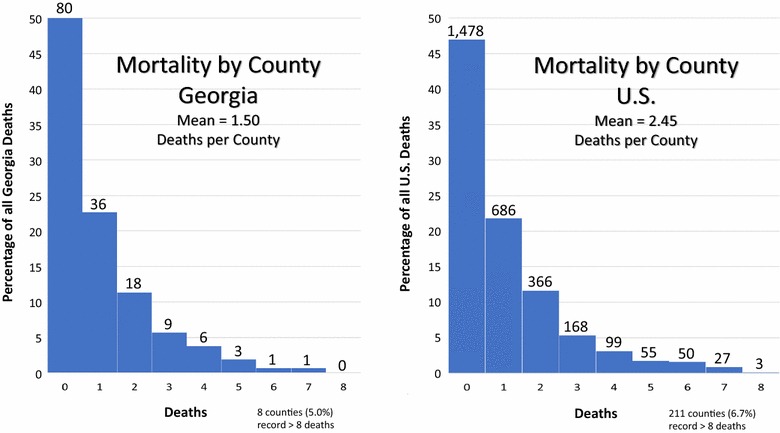
Distribution of adolescent cancer county mortality counts. Adolescent cancer mortality counts from the GADPH were appropriate for testing the methods. The distribution of county mortality counts for Georgia mirror those of the U.S. Likewise, patterns of zone values are roughly similar for the state and the nation

Histograms of the distribution of county mortality counts reveal a pattern in Georgia similar to that of the U.S. (Fig. 6). The histogram of the Georgia mortality counts (N = 238) demonstrates a Poisson distribution, strongly right skewed. Of 159 counties, 80 (50%) record zero mortalities for the 13-year period. Seventy-two counties (45%) report between one and five deaths and fewer than 5% of counties (n = 7) record more than five deaths. The mean number of deaths by county for Georgia is 1.50. The histogram of the U.S. mortality counts (N = 7687) demonstrates a Poisson distribution, strongly right skewed. Of 3143 counties, 1478 (47%) record zero mortalities for the 13-year period. Forty-four percent of counties (1374) report between one and five deaths and 9% of counties record more than five deaths (n = 291). The mean number of deaths by county for the U.S. is 2.45.

### Transformation error: degrees of hierarchy and fit

As discussed above, the degree of hierarchy (nesting) and the degree of fit (overlap) are two measures to express the amount of estimation, or error, involved in the transformation from source to target zones, particularly affecting the cartographic methods. The closer the output of either of these measures to 100%, the better the transformation estimate should be. Table 1 shows the degrees of hierarchy and fit, in percentages, for both the Georgia and U.S. denominators, which use census tract source zones for populations, and numerators, which use county source zones for numbers of deaths. Denominator percentages for hierarchy, and particularly for fit, are high, with overall hierarchy at 81.7% for Georgia and 83.7% for the U.S., and overall fit at 96.6% for Georgia and 97% for the U.S. Numerator percentages for all measures are much lower than those for denominators, meaning the error is higher for numerator estimation. Overall hierarchy is 52.2% for Georgia and 45.1% for the U.S. Overall fit is 88.7% for Georgia and 87.2% for the U.S. Of note is the zone A degree of hierarchy for Georgia; a zero value means that none of the counties nest completely within zone A. Patterns of zone values are roughly similar for Georgia and the U.S. For example, most zone D measures indicate less potential for error than those of the other zones, because it is large relative to other zones, with little change-of-support.

**Table 1:**
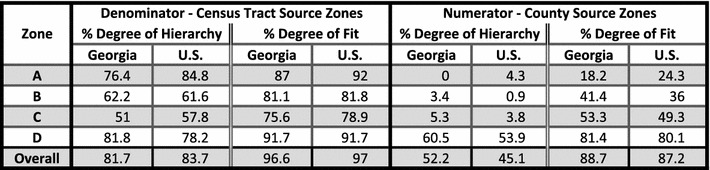
Measures of potential error: degrees of hierarchy and fit

Degree of hierarchy (nesting) and degree of fit (overlap) between source and target study zones. The higher the percentage, the better the estimate

### Comparisons between observed and estimated mortality measures

Table 2 shows comparisons between observed 1999–2011 Georgia adolescent cancer mortality and estimated mortality, by method and zone. For the death counts (i.e., numerators), the “Georgia total” row illustrates the concept of volume preservation. That is, each of the four cartographic methods maintained overall counts, unlike the geostatistical method. The arithmetic differences between the observed counts and those for the methods become apparent in the zone estimations. The mean absolute value arithmetic differences between the observed Georgia mortality counts and their paired count estimates, were 5.50, 5.00, 4.17, 2.84, and 3.43 for each of the five methods, respectively. Standard deviations of these means decrease progressively for the cartographic methods 1 through 4. Geostatistical method 5, however, has a standard deviation higher than method 4, but slightly lower than method 3. The largest absolute arithmetic difference for method 4 was less than five, whereas for methods 1, 2, 3, and 5, the largest arithmetic differences were much greater, at 16, 11, 8.59, and 7.85, respectively. Comparing the methods through paired t-tests of absolute value arithmetic differences, however, showed no statistical difference among the methods, with no method a statistically significantly closer estimator than any other method.

**Table 2:**
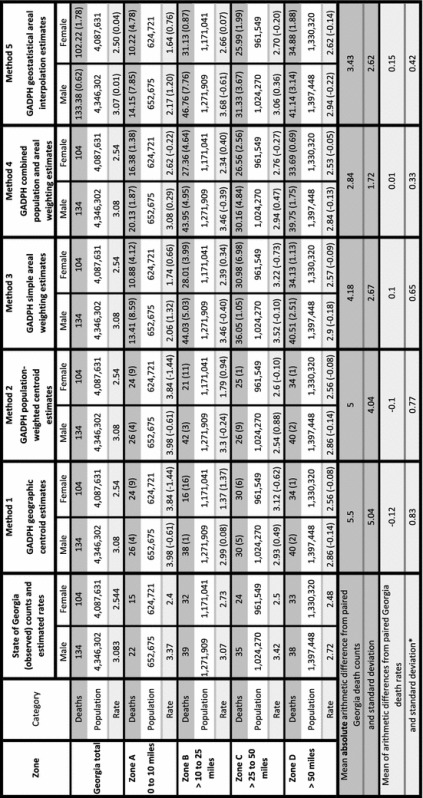
Comparisons between observed 1999–2011 Georgia adolescent cancer mortality and estimated mortality, by method and zone

Rate per 100,000 per year

1999–2011 person-year population estimated using simple areal weighting with 2000 and 2010 tract-level source zones, then weighted by year

Methods 3 through 5 use death estimates expressed as real numbers, in contrast to the centroid methods which use integer counts

Value in parentheses is the arithmetic difference between the estimate and its paired state of Georgia value

We use absolute arithmetic differences for death counts because if we used actual differences, the mean difference would equal 0

**Fig. 7:**
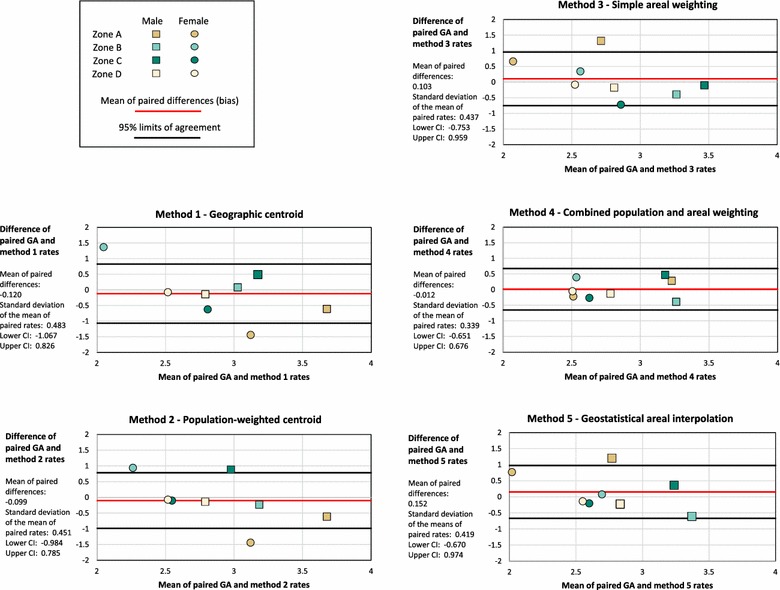
Bland–Altman plots to compare Georgia rates with the five method rates. The Bland–Altman plots compare 1999–2011 Georgia adolescent mortality rate estimates to estimated rates for methods 1 through 5. Method 4 demonstrates the greatest agreement

*Standard deviations of the mean arithmetic difference from paired Georgia deaths rates are distinct from those shown on Fig. 7, which are standard deviations of the mean of the paired rates

Table 2 also displays the robust denominator estimates as well as rates by method and zone. The mean of arithmetic differences from paired Georgia death rates are −0.12, −0.10, 0.10, 0.01, and 0.15 for the methods, 1 through 5, with method 4 closest to zero and method 5 furthest from zero. As with the counts, the standard deviations of these means decrease progressively for the cartographic methods, with method 4 the lowest at 0.33. For method 5, however, the standard deviation of the mean of the arithmetic differences from paired Georgia rates, at 0.42, falls between those of methods 3 and 4.

We calculated the Pearson product moment correlation coefficients (Pearson’s r) for the rates. For methods 1 through 5, the r values were 0.184, 0.191, 0.327, 0.627, and 0.413 respectively. In social science research, methods 1 and 2 demonstrate weak positive correlations, methods 3 and 5 suggest moderate positive correlations, and method 4 a strong positive correlation with the Georgia rates.

For each of the five methods, we used Bland–Altman plots, a tool to compare methods estimating the same variable, to visualize the agreement between arithmetic differences of paired Georgia and method rates (Fig. 7). Usually Bland–Altman plots measure equipment performance against a known standard. We apply them here to assess geographic data processing methods as compared to known data values. The plots display the means of each pair of rate estimates (x value), versus the arithmetic differences between the paired estimates (y value). For example, the estimated Georgia mortality rate for males in zone A is 3.371, whereas for method 1 the estimated rate is 3.984 (see Table 2). The mean of these values is 3.667 and the difference is −0.613. This point (3.667, −0.613) is displayed as the rightmost square on the method 1 plot of Fig. 7. The plots also display the mean of the arithmetic differences between the Georgia estimates and each paired estimate, known as the bias, as a red horizontal line. Limits of agreement, confidence intervals at the 95% confidence level, are drawn as black lines. For the method to be a good match with the Georgia estimated rates, all the plotted points must fall within the limits of agreement, close to the bias. Of the five plots, method 4 most closely replicates the Georgia estimates; all the plotted points are within the limits of agreement, which is also the smallest of the five methods, and the mean of arithmetic differences is closest to zero.

## Discussion

Among the five methods tested for numerator estimation, method 4, the combined population and areal weighting technique, had the lowest mean absolute value arithmetic difference between the estimation and observed Georgia death counts. Method 4 also generated the only strongly positive correlation with the estimated Georgia rates. However, correlation tests, i.e. Pearson’s r, which support the selection of method 4 as the best method, are inadequate to completely assess the accuracy of an estimation method. A strong correlation may exist, but the output measurements could, theoretically, be consistently different. A more definitive measure of method performance is that of agreement. To visualize agreement, we used Bland–Altman plots which display the means of each pair of estimates—the Georgia rates compared to each of the five methods—against the arithmetic difference between the estimates. Method 4 again produced the best results, with each of the eight plotted points falling within small 95% limits of agreement.

Examining the other methods, we observe several reasons for their weaker performance. Although method 1 is easy to perform, the county centroid location is based solely upon the county’s geographic center of gravity, with no accounting for the distribution of the study populations. This binary “all or nothing” condition means that mortality assignment could be 100% incorrect (or 100% correct or any percentage in between). Method 1 therefore returned the least accurate results. Population-weighted centroid assignment, method 2, improved centroid placement, but was still limited by the binary nature of the potential error as exemplified in method 1. The two centroid methods generated the highest absolute arithmetic differences from the Georgia counts, weak positive correlations with the Georgia rates, and displayed—via Bland–Altman plots—a lack of agreement with Georgia rates. Method 3, simple areal weighting, is superior to the centroid methods, indicating an intermediate absolute value difference from Georgia counts as well as a moderate positive correlation with the Georgia rates. However, method 3 failed the agreement test, most likely because the affected population was not taken into account inasmuch as simple areal weighting assumes an evenly distributed population.

Geostatistical areal interpolation, method 5, showed slightly stronger positive correlation with the Georgia rates than method 3. However, the geostatistical method still failed the agreement test. This lack of agreement may be the result of the nonstationary nature of the source data. Mortality count data should vary in a similar way to population, which is known to be somewhat nonstationary. The violation of the stationarity assumption makes fitting model parameters much more difficult, and limits the accuracy of the probability surfaces produced. In addition, geostatistical areal interpolation does not preserve volume, as do the cartographic methods tested.

There is also a conceptual problem with method 5. Count data are inherently discrete rather than continuous. As geostatistical methods are surface generating, i.e. they create continuous data, the use of geostatistics to interpolate counts is tenuous. While we would have preferred to interpolate mortality rates, the high number of counties with zero mortalities (80 of the 159 Georgia counties had no adolescent cancer deaths during the time of the study) precluded rate interpolation as the model invariably assigned a rate of zero across the study region. However, in the case of event interpolation, the data structure mismatch is solved by producing a continuous probability surface, rather than a prediction surface, from which to estimate COG zone counts. The surface generated represents the probability of an event occurring based upon the number of times that event occurred in each of the original geographies, mortality count by county in this study. This type of interpolation may be problematic if something other than the underlying distribution of counts affects the probability of observing the event, e.g. if different counties had different reporting practices.

Method 5 also presented a unique challenge that could makes its application difficult for those without expert knowledge of geostatistical methods. Aside from the difficulty associated with the visual variography required when using the Geostatistical Wizard in ArcMap, geostatistical areal interpolation can be sensitive to data structuring. For this project, shapefiles used for the COG target zones had to be preprocessed so that aggregation of the probability surface to the target zones would produce accurate results. Specifically zone D, shown in Fig. 1, posed a problem. In the state of Georgia zone D encompassed an area of roughly 103,000 km^2^, whereas the next largest zone covered only about 15,000 km^2^. Although this large land expanse with little change-of-support produces good results for the cartographic methods, the size disparity led to the over estimation of mortality counts and the prediction of a high standard error in zone D when the geostatistical probability surface was aggregated to the COG study zones. To reduce predicted error, we split zone D into nine smaller polygons, bringing the largest individual polygon down in size to roughly 18,000 km^2^ and reducing the predicted standard error for male mortality counts from 40.99 in the combined zone D to a mean of 3.62 and sum of 32.59 for the nine polygons that make up zone D. Female count corresponding standard error numbers were 35.68, 3.16, and 28.42 respectively. Summing the estimated counts in these nine zones provided reasonably accurate results, shown in Table 2, especially as compared to the estimated counts when zone D was not split (77.86 for males, 56.82 for females). We expect this size disparity between zone D and the other study zones to require even more preprocessing for a national scale geostatistical analysis.

The most effective method, method 4, incorporated ancillary census tract data to weight deaths by the at-risk populations to estimate mortality, the intent being to reduce the error associated with assuming an evenly distributed population across county source zones. In essence, disaggregation using population weighting is analogous to locally fitting the distribution of each source zone. Additionally, in combined population and areal weighting, unlike centroid methods or simple areal weighting, error is distributed across the target zones by allocating “mortality” weighted by population and area. Although it is more processing-intensive than the other cartographic methods described here, the processing can be automated. Further, method 4 is conceptually simple, particularly in contrast to the geostatistical techniques of method 5.

All spatial disaggregation techniques generate error. Because of confidentiality requirements, we were limited to county resolution for the NCHS numerator mortality data as opposed to tract-level resolution for the denominator populations. Denominator estimation was straightforward and stable because the tract source zones were small relative to the larger target zones surrounding the COGs, the degrees of hierarchy and fit were large, and the populations large.

In contrast, numerator estimation was more challenging. The Wilson and Mansfield population-weighting technique, which informed the population-weighting component of our combined population and areal weighting method, transformed mortality rates from one set of standard zones (counties) to another set of standard zones (congressional districts) both built from perfectly nested census blocks with 100% hierarchy and fit. In contrast, we required numerator mortality counts to be transformed from counties to non-standard study zones. We therefore combined population, in a conceptually dasymetric approach, and areal weighting, to estimate numbers of deaths for the numerators of our study zones.

Source zones for the numerator were counties within which census tracts nest hierarchically. Counties were therefore, by definition, larger than the tract source zones used for denominator estimation, with the rare exception of counties consisting of a single tract. Lower degrees of hierarchy and fit reflect this dichotomy between counties and tracts. Small numbers of deaths per county also led to less stable results for numerator estimation. In sum, low hierarchy and fit values for the numerators, along with smaller numerator counts, showed greater error in numerator estimation, in contrast to the high hierarchy and fit measures, as well as much larger counts, for the denominators.

Adolescent cancer mortality counts from the GADPH were appropriate for testing the methods explored. The distribution of county mortality counts for Georgia mirror those of the U.S. Likewise, patterns of zone values are roughly similar for the state and the nation. In terms of area, however, medium-sized Georgia has some of the smallest counties in the country (N = 159) and therefore may not be representative of other U.S. states. As noted, the mean number of mortalities per county is 1.50 versus 2.45 for the U.S. as a whole. It may be that counties with smaller geographic areas return better results than larger counties for the five tested methods. However, as method 4 employs combined weighting, which distributes error across study zones, we would still expect to observe improved estimation over the centroid methods in regions of the country with larger counties. With Georgia’s smaller counties, improvements over the other methods in this study should be seen as conservative.

One potential limitation involves the relationship between census tract population and geographic area. The optimal population for a tract is 4000, therefore less densely populated counties are likely to have fewer tracts, though with larger geographic areas. Georgia counties have higher population densities and smaller tracts than many counties in other states, so error cannot be distributed at as fine a level of granularity elsewhere as in Georgia. For our own primary research, however, counties with small numbers of tracts were not a major concern because those counties are located in zone D, which has limited change-of-support.

Another limitation was the small number of statistical data points available, eight (four zones by two sexes) for each method. Examining these four methods in other states would provide additional data points along with an opportunity to study the effects of larger or less densely populated counties on estimation methods. Another approach to increase statistical data points for method validation would be to explore Bland–Altman plots of additional zone configurations within the state of Georgia, e.g. random region delineations.

We chose not to examine regression to estimate mortality because the purpose of the primary study was solely to examine the association between adolescent cancer mortality and distance to a COG. Other than population distribution by sex, we avoided a priori assumptions in our estimation of the COG proximity zone mortality patterns. We also wanted to avoid the complexities of U.S.-wide regression models using multiple covariates. Given the satisfactory results we obtained from population and areal weighting, simple in concept and practice, we did not see the need to include multivariate regression in our preliminary analysis. Nonetheless, race, ethnicity, poverty, and lack of health insurance, among other factors, influence adolescent cancer mortality distribution. These factors vary geographically and will be considered in future exploration of potential explanatory variables in the primary study.

## Conclusions

This research demonstrates that combined population and areal weighting, compared to cartographic centroid and simple areal weighting methods, and a geostatistical method, returns more accurate estimates of mortality in transforming small counts by county to aggregated counts for large target zones that do not conform to standard enumeration units. Weighting by ancillary population data to take into account at-risk population, in conjunction with the allocation of weighted mortalities, which eliminates the “all or nothing” problem inherent in centroid methods, distributes error across study zones, thus improving estimates. Furthermore, practitioners without the resources of geospatial statisticians and software, may find this simpler cartographic method more accessible and just as effective in transforming county-level source zone counts to larger, non-standard target zones. This methodology should be of interest to practitioners and researchers limited to analysis of count data for relatively large enumeration source units, such as NCHS county-level mortality counts, among other data sources. We expect to observe increased support for using combined population and areal weighting estimates, particularly over other cartographic overlay methods.

## Abbreviations

CD: congressional district
CI: confidence interval
CMF: Compressed Mortality File
COG: Children’s Oncology Group
NCHS: National Center for Health Statistics
GRASP: Geospatial Research, Analysis, and Services Program
GADPH: Georgia Department of Public Health
GA: Georgia

## Footnotes

1 Although NCHS CMF users are permitted to estimate sub-national counts and rates for their own analyses, they cannot report any sub-national count or rate based on totals less than 10. NCHS CMF users, as well as users of any confidential data sets, must ensure they comply with data use agreements.
